# The feasibility of implementing an exercise programme for deconditioned cancer survivors in a national cancer centre: FIXCAS Study

**DOI:** 10.12688/hrbopenres.12925.2

**Published:** 2020-12-18

**Authors:** Kate Devenney, Niamh Murphy, Ronan Ryan, Clíona Grant, John Kennedy, Rustom P. Manecksha, Orla Sheils, Margaret L. McNeely, Juliette Hussey, Grainne Sheill

**Affiliations:** 1 University of Dublin, Dublin, Ireland; 2 St. James's Hospital, Dublin, Dublin, Ireland; 3 University of Alberta and Cross Cancer Institute, Edmonton, Canada

**Keywords:** Exercise, Rehabilitation, Cancer

## Abstract

**Introduction:** As both the number of cancer survivors and the length of survival time are increasing, long-term health issues related to cancer and its treatment are becoming more prevalent. Research suggests that exercise can mitigate several negative health consequences in cancer survivors and improve physical function and quality of life. Multi-modal exercise interventions have been proposed as a cornerstone for survivorship care. However, studies evaluating exercise programmes within the Irish population are lacking.

**Purpose:** To evaluate the introduction, implementation and acceptability of a multi-modal exercise rehabilitation programme for deconditioned cancer survivors in a real-world, standard practice setting.

**Methods and analysis:** In this single-arm prospective feasibility study, cancer survivors (n=40) will undergo a 10-week multi-modal exercise programme. The study population will comprise of cancer survivors attending outpatient services in an Irish national cancer centre. Participants will be aged 18 or older and have completed treatment with curative intent. Feasibility will be evaluated in terms of recruitment, adherence and compliance to the programme. Secondary outcomes will examine physical function and quality of life measures. In addition, the acceptability of the programme will be assessed through stakeholder feedback.

**Ethics and dissemination**: Ethical approval through the St. James’s Hospital and Tallaght University Hospital Research and Ethics Committee is currently pending. The study results will be used to optimise the intervention content and may serve as the foundation for a larger definitive trial. Results will be disseminated through peer-review journals, congresses and relevant clinical groups. **Trial registration**: ClinicalTrials.gov NCT04026659 (19/07/19)

## Introduction

There are currently more than 150,000 cancer survivors in Ireland, and this number continues to rise ( Department of Health, 2017). Furthermore, with advances in early detection and treatment of cancer in the context of an aging population, by 2020 1 in 2 Irish people will be a cancer survivor ( Department of Health, 2017). Subsequently, as both the number of cancer survivors and the length of survival time are increasing, long-term health complications related to cancer treatment are becoming more prevalent ( Miller **et al**., 2016; Rowland & Bellizzi, 2014).

Depending on treatment pathways, cancer survivors can face several negative consequences of cancer treatment that include psychological (depression or anxiety, fear of recurrence, cognitive impairment) and/or physical symptoms (pain, peripheral neuropathy, sexual dysfunction, gait and balance deficits, joint mobility issues and lymphoedema) ( Beesley *et al*., 2007; Caraceni & Portenoy, 1999; Hendren *et al*., 2005; Massie, 2004; Mehnert *et al*., 2009; Mock *et al*., 2001; O¢Dell & Stubblefield, 2009; Quasthoff & Hartung, 2002; Vardy *et al*., 2007). Numerous systematic reviews demonstrate that exercise can mitigate a number of these factors in cancer survivors and improve quality of life, fatigue, physical function and cardiorespiratory fitness and can optimise functional status, preserving the ability to remain in the workforce and fulfil other life roles ( Courneya & Friedenreich, 1999; Galvão & Newton, 2005; McNeely *et al*., 2006).

Despite the robust body of existing literature, the integration and delivery of exercise rehabilitation and survivorship into standard clinical cancer care continues to remain the exception rather than the norm ( Mulcahy *et al*., 2018). Internationally, models of cancer survivorship care have been developing rapidly in recent years, many centring on the provision of exercise rehabilitation programmes across diverse delivery settings ( Oeffinger & McCabe, 2006). However, referral to exercise specialists is not a part of the standard care received by oncology patients in Ireland, with a distinct lack of rehabilitation services available for cancer survivors ( Mulcahy *et al*., 2018). There is a need to investigate the feasibility of delivering exercise-based rehabilitation to patients completing cancer treatment.

The aim of the FIXCAS (The Feasibility of Implementing an eXercise programme for deconditioned CAncer Survivors in a national cancer centre) study is to examine the feasibility of implementing a 10-week multi-modal exercise rehabilitation programme to deconditioned cancer survivors in a National Cancer Centre. The implementation of the programme inform the integration of exercise rehabilitation into survivorship services in Ireland.

## Methods

### Study aim

The overall aim of this work is to examine the feasibility of implementing a 10-week multi-modal exercise programme for cancer survivors. Feasibility will be determined by the following outcomes; recruitment, adherence and retention rates, acceptability of the programme and any adverse events.

Secondary aims are:

- To examine the effect of the FIXCAS programme on physical function.
- To examine the effects of the FIXCAS programme on patient reported outcomes including HRQOL and fatigue.
- To examine the costs associated with a ten-week exercise intervention in the cancer setting.

### Study design

This is a single-arm prospective feasibility study for deconditioned cancer survivors in a real-world, standard practice setting. A convenience sample of patients (n=40) attending outpatient oncology services in St James’s Hospital, a National Cancer Centre, will be recruited. This centre does not currently have an exercise-based rehabilitation service for cancer survivors. This study will primarily assess feasibility of the exercise intervention and the data from the pilot trial will be used to inform a sample size calculation for a definitive randomised controlled trial.

### Study population

To be eligible to participate in this study, an individual must provide a signed consent form and meet the following eligibility criteria: 18+ years old, diagnosis of solid tumour, completion of chemotherapy and/or radiotherapy and/or surgery with curative intent within the preceding 12 months. All patients must receive medical clearance from their medical team before participating in the study. Only patients who presented with a self-reported loss of functional capacity, including a loss of physical fitness or muscle tone or a decrease in physical activity levels were considered deconditioned and eligible for participation ( Broderick *et al*., 2013). Individuals with moderate or severe cognitive impairment, currently pregnant or receiving treatment in the palliative setting will be excluded from participation in this study.

### Recruitment

Potential participants will be recruited by direct invitation from study personnel in oncology clinics, by recommendation from medical or multidisciplinary colleagues, or by responding to mail-out of a study leaflet (sent out to individuals consenting to mail-out of information). Informed consent will be obtained in writing from participants by designated members of the research team (Extended data ( Sheill, 2019)).

### Intervention

The FIXCAS multi-modal exercise programme is designed in accordance with international guidelines for best practice exercise prescription for people with cancer ( Schmitz *et al*., 2010). The FIXCAS exercise programme is theoretically underpinned by the Theory of Planned Behaviour, the most widely used theory of exercise motivation for people with cancer ( Armitage & Conner, 2001; Rhodes & Courneya, 2003). The intervention consisted of three motivational techniques designed to translate intentions into behavior. Patients set weekly goals for their home-based exercise and also received an exercise information booklet from the research team (instruction on how to perform the behavior). Patients also received feedback on their weekly physical activity from the research team during weekly exercise sessions.

**Figure 1.**
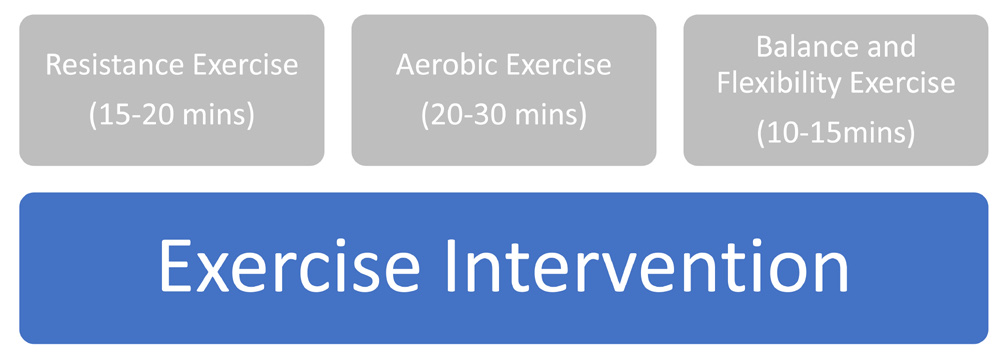
Outline of Exercise Intervention.

The exercise programme includes 10 weeks of twice weekly group-based exercise sessions administered under the supervision of a physiotherapist with extensive training in the area of oncology. Each exercise session will last approximately 1 hour and consist of a combination of aerobic, resistance and balance and flexibility exercises ( Figure 1).

***Aerobic exercise*** will consist of 20 to 30 min of moderate intensity cardiovascular exercise using a variety of modalities such as walking or jogging on a treadmill, cycling or rowing on a stationary ergometer. Patient’s baseline activity levels, exercise preferences and physical impairments will inform the choice of exercise modalities. Heart rate will be monitored using Polar FT7 heart rate monitors (Polar Electro, Lake Success, NY) and the BORG Rating of Perceived Exertion ( Borg, 1998). Participants will exercise to a target intensity of 40–70% of estimated heart rate reserve (HRR) or 12–15 on the BORG Rating of Perceived Exertion (RPE) scale. Aerobic exercise will commence at a low intensity (40–55% HRR) and progress to moderate intensity (55–70% HRR) over the 10-week programme.

***Resistance exercise*** will target the large muscle groups of the upper and lower extremities be performed at 40% to 70% of the measured one repetition maximum (1-RM) and consist of two sets of 10–15 repetitions. Resistance exercise will commence at a light to moderate intensity and will progress to a moderate intensity over the 10-week programme. Eight major upper limb and lower limb muscle groups will be exercised eg. biceps curl, leg extension.

***Flexibility and balance exercise*** will be incorporated into the two supervised weekly sessions, as per current guidelines.

***Self-directed care:*** A home exercise programme is included as a self-managed component of the programme which aligns with national recommendations for survivorship care. This self-management component is included to improve compliance and stimulate physical activity outside the exercise programme. Patients will be encouraged to be moderately physically active for at least 30 minutes, three times per week in addition to the supervised programme in order to meet current physical activity guidelines ( Schmitz *et al*., 2010).

***Maintenance of the exercise intervention:*** All patients completing the FIXCAS programme will receive a written exercise summary upon completion (extended data ( Sheill, 2019)) of the programme to facilitate transition to a local community exercise setting.

### Outcomes

Outcomes will be assessed at baseline (T1) and at the completion of the 10-week intervention (T2). A follow up Quality of Life (QoL) Questionnaire (EQ-5D-5L) will be posted to participants 3 months postintervention (T3). Table 1 outlines the schedule of study assessments. A member of the research team will collect quantitative measures across all study time points. The qualitative element of the study will be undertaken after completion of the exercise programme.

**Table 1.** Outline of study assessments. EQ-5D-5L: EuroQol-5D-5L; IPAQ: International Physical Activity Questionnaire; SPPB: Short Physical Performance Battery; 1-RM: 1 Repetition Maximum; EORTC-QLQ-C30: European Organisation for Research and Treatment of Cancer- Quality of Life Questionnaire-C30.

Timepoints		Pre-Screening Telephone Call	T1 Pre-intervention	10-week intervention	T2 Post Intervention	T3 3 months post intervention
**Initial Study** **Procedures**	Explain study	X	X			
	Screen eligibility criteria	X	X			
	Sign consent form		X			
	Case report form and clinical data		X			
**Feasibility** **Outcomes**	Enrolment rates		X			
	Adherence			X		
	Adverse Events			X		
	Retention rates			X	X	
	Stakeholder feedback					X
**Secondary Study** **Outcomes**	EQ-5D5L		X		X	X
	Cost analysis of programme					X
	Six-minute walk test		X		X	
	IPAQ		X		X	
	SPPB		X		X	
	1-RM		X		X	
	EORTC-QLQ-C30		X		X	
	Brief Fatigue Inventory		X		X	
	Body composition		X		X	
	International Consultation on Incontinence Questionnaire (Prostate cancer only)		X		X	
	Brief Male Sexual Function Inventory (Prostate cancer only)		X		X	

***Primary outcome.*** The primary outcome of this study will be its feasibility aspects, including recruitment rates (percentage of eligible study population that consented to participation), programme adherence (number of prescribed supervised and unsupervised sessions completed), retention, acceptability of the intervention and adverse events. Reasons for poor enrolment, attrition or non-compliance will be identified through qualitative evaluation with participants and medical professionals upon programme completion.

***Secondary outcomes.*** Several secondary outcomes will investigate the impact of the intervention on physical function and QoL. Physical fitness will be measured by the 6 minute walk test (6MWT), a valid and reliable measure of physical fitness in people with cancer which will be performed according to the American Thoracic Society (ATS) Guidelines ( American Thoracic Society, 2002; Schmidt *et al*., 2013). Self-reported physical activity will be collected using the International Physical Activity Questionnaire (IPAQ) ( Hagströmer *et al*., 2006). Physical Performance will be measured by a Short Physical Performance Battery (SPPB) and a lower limb strength test (Leg extension 1-Repitition Max).

Quality of life is evaluated through the internationally established European Organisation for the Research and Treatment of Cancer Core Quality of Life Questionnaire (EORTC-QLQ-C30) ( Aaronson *et al*., 1993). It assesses important functioning domains (e.g. physical, emotional, role) and common cancer symptoms (e.g. fatigue, pain, nausea/vomiting, appetite loss) ( Aaronson *et al*., 1993). Euro-QoL (EQ-5D5L) a generic quality of life measure will also be used ( Herdman *et al*., 2011). This measure will be completed by all participants and will form part of the cost analysis of the programme. Fatigue will be assessed using the Brief Fatigue Inventory (BFI) ( Mendoza *et al*., 1999). Prostate cancer survivors will complete questionnaires on incontinence (International Consultation on Incontinence Questionnaire) ( Hajebrahimi *et al*., 2004) and sexual function (Brief Male Sexual Function Inventory) ( O¢Leary *et al*., 1995). Both incontinence and sexual function may be affected by the exercise intervention ( Baumann *et al*., 2012).

A needs assessment (impairment screening) of each individual will be collected at T1 and T2 study assessments. Body composition will also be collected at T1 and T2. Weight (kilogrammes (kg)) and height (centimetres (cm)) will be recorded by standard methods and body mass index (BMI) will be calculated as weight (kg)/ height (metres (m ^2^)). Adverse outcomes will be recorded throughout the study period.

***Qualitative evaluation.*** Acceptability of the intervention will be explored through qualitative interviews. Key stakeholders, namely participants in the exercise intervention and health professionals referring to the exercise programme, chosen at random, will be invited to participate in semi-structured feedback interviews. Open-ended questions will be used to encourage open dialogue and elaboration on different aspects of the programme. Health professionals (n=8-10) referring to the programme will be interviewed to examine their experience of referring to the FIXCAS programme to identify barriers and facilitators to referral and to determine areas for review and further development. Patients (n=10-15) will be asked to evaluate satisfaction with the intervention. The interviews will be audio recorded and transcribed, following which data analysis of the interviews will occur through content analysis using simple descriptive thematic analyses.

## Safety

All serious adverse events (SAE)/adverse events (AE) will be recorded on study specific adverse event forms. All AEs will be registered with the principal investigator (PI). These will be discussed at regular team meetings and collected and registered at the end of the study. In the case of an SAE, the PI will be informed at time of occurrence (with 24 h). The investigator can decide to withdraw a participant from the study for urgent medical reasons or safety concerns. Participants can leave the study at any time for any reason if they wish to do so without any consequences. Participants who withdraw from the study will be invited to attend assessment.

## Data management

Data will be collected and recorded in a paper-based study case report form (extended data ( Sheill, 2019)). The case report form will maximise the quality of the data captured and minimise the risk of erroneous data collection. Each case report form will be assigned the participant study identification code to ensure patient anonymity. Qualitative interviews will be recorded using a dictaphone and transcribed. Coded information will be stored on a secure, password protected, encrypted computer. The key to the participants code will be stored separately in a password protected file, on a secure, password protected, encrypted computer at the study site. All hard copies of study data, electronic interview transcripts and audio recordings will be retained and stored securely for 10 years at Trinity College Dublin.

The data repository will be maintained by designated members of the research team who will input data. Data will be only be accessible to authorised members of the research team. All authorised team members will receive training regarding the data management plan before authorisation is granted for data processing. In line with open access publication requirements, the anonymised final data set will be archived in a secure online data repository.

## Sample size and statistical analysis

A sample of 40 participants will be recruited to determine the feasibility of the FIXCAS programme. As this is a feasibility study, a sample size calculation was not performed however similar sample sizes have been utilised in other feasibility trials in cancer survivorship ( Quist *et al*., 2012).

Data analysis will be performed using IBM SPSS Statistics version 24 (SPSS Inc, Chicago, IL). Summary statistics for continuous variables (means and standard deviations or medians and ranges as appropriate) and categorical variables (counts and proportions) will be presented. Graphical summaries will be used to compare the distributions of each response variable and for patient characteristics. A linear mixed model will be used to model the longitudinal change in the primary measures while adjusting for the response variable and for the within subject correlation in the repeated measures across time.

## Ethics and dissemination

Ethics approval will be granted by St. James’s Hospital and Tallaght University Hospital Research Ethics Committee (approval pending). The study results will be used to optimise the intervention content and may serve as the foundation for a larger definitive trial. We aim to disseminate the results through peer-review journals, presentation at conferences and relevant clinical groups. The International Committee of Medical Journal Editors (ICJME) recommendations will be adhered to in all reporting of trial data.

## Study status

The study is currently pending local Research and Ethics Committee approval and is not yet recruiting.

## Discussion

The article describes the protocol of a feasibility study evaluating an individualised 10-week FIXCAS multi-modal exercise programme in deconditioned cancer survivors aiming to improve physical function and quality of life.

Research has demonstrated that cancer survivors experience physical deficits including low levels of physical activity, poor cardiorespiratory fitness ( Gannon *et al*., 2017) and sarcopenia ( Elliott *et al*., 2017). As physical fitness has been broadly linked to the quality of life of cancer survivors, fitness can be considered a modifiable factor that can subsequently impact positively on quality of life ( Cheema & Gaul, 2006; Courneya *et al*., 2003; O’Neill *et al*., 2018).

Despite robust evidence supporting the role of exercise in cancer recovery, none of the eight cancer centres in Ireland provide exercise based survivorship programmes for cancer survivors and exercise rehabilitation is not an element of standard care for patients with cancer in Ireland ( Mulcahy *et al*., 2018). High feasibility and acceptability of exercise interventions in a research setting has been demonstrated for oesophageal cancer survivors in Ireland. A 12 week supervised and home- based exercise and education sessions resulted in clinically significant improvements in functional performance and QoL ( O’Neill *et al*., 2017). However, the feasibility of offering an exercise rehabilitation programme to a broad range of cancer survivor groups in the clinical setting of a national cancer centre requires further exploration.

The intervention consists of a 10 week (2 sessions weekly) individualised multi-modal exercise programme. The content of the intervention was modelled on national and cancer-specific recommendations of the American College of Sports Medicine and evidence from existing literature and guidelines ( Schmitz *et al*., 2010). The American College of Sports Medicine (ACSM) has concluded that exercise both during and after cancer treatment is safe and should be encouraged, although prescriptions should be individualised according to the patient ( Schmitz *et al*., 2010). Therefore, the FIXCAS programme will be tailored to each individual patient taking into consideration their current health status, physical activity levels, exercise preferences and individual post treatment impairments following the ACSM recommendations.

Important strengths of the intervention include the application of the programme in a real-world clinical practice setting. Secondly, we consider the timing of the intervention to be advantageous. Surveys of cancer survivors clearly show a preference for commencing an exercise program after primary treatments have been completed, with many studies indicating a preference for the 3–6-month period after completion of treatment ( Broderick *et al*., 2013). This time period, termed the ‘recovery or rehabilitation period’, may be the optimal window for commencing an exercise program to reverse a downward trajectory in activity levels and fitness as well as addressing any lingering treatment-related side effects ( Broderick *et al*., 2013).

## Conclusion

In this article, we present the study design to investigate the feasibility of delivering a 10-week multi-modal exercise rehabilitation programme in a national cancer care centre. In addition, we outlined the protocol of an intervention aimed at improving physical fitness, quality of life and other health related outcomes in cancer survivors. The results of the feasibility study may be used for optimisation of the intervention content and may serve as a foundation for evaluating the intervention in a larger randomised controlled trial.

## Ethics approval

St. James’s Hospital and Tallaght University Hospital Research and Ethics Committee (approval pending). Any protocol modifications will be communicated in writing to the Research and Ethics Committee and updated on the trial registration.

## Data availability

### Underlying data

No data are associated with this article

### Extended data

Data Archiving and Networked Services (DANS): The Feasibility of Implementing an Exercise Programme for Deconditioned Cancer Survivors in a National Cancer Centre: FIXCAS Study: https://doi.org/10.17026/dans-26u-qejk ( Sheill, 2019)

This project contains the following extended data:

- FIXCAS Patient Consent_Version2 290819.pdf
- FIXCAS Completion summary v1 2.8.19.pdf (exercise programme completion summary to facilitate transition to community based exercise)
- FIXCAS CASE REPORT FORM Draft Version 2.0 31.7.19.pdf (study case report form including outcome measures)

### Reporting guidelines

SPIRIT checklist for ‘The feasibility of implementing an exercise programme for deconditioned cancer survivors in a national cancer centre: FIXCAS Study’, https://doi.org/10.17026/dans-26u-qejk ( Sheill, 2019)

Data are available under the terms of the Creative Commons Zero “No rights reserved” data waiver (CC0 1.0 Public domain dedication).
