# Supplementary material for: The feasibility of implementing an exercise programme for deconditioned cancer survivors in a national cancer centre: FIXCAS Study
Source: HRB Open Res. 2020 Dec 18;2:24. Originally published 2019 Sep 30. [Version 2] doi: 10.12688/hrbopenres.12925.2 (PMC8030104; doi:10.12688/hrbopenres.12925.2)
Supplement: Peer review history

# Peer review: Reviewer response for version 2

**DOI:** 10.21956/hrbopenres.14333.r28555

**Published:** 2021-04-06

**Authors:** Brian McGuire

**Affiliations:** 1 School of Psychology, Centre for Pain Research, National University of Ireland, Galway, Galway, Ireland

The authors have addressed the main issues and recommendations of the reviewers.

 A few minor suggestions (but no need for further review):

1. The title should reflect that this is a protocol paper.
2. The revised sections of text in relation to method/procedure have been written in past tense whereas the rest of the method is in present or future tense (protocols are usually written in future tense). This will need to be revised.

Is the study design appropriate for the research question?

Yes

Is the rationale for, and objectives of, the study clearly described?

Partly

Are sufficient details of the methods provided to allow replication by others?

Partly

Are the datasets clearly presented in a useable and accessible format?

Not applicable

Reviewer Expertise:

Psychosocial interventions for chronic physical illnesses.

I confirm that I have read this submission and believe that I have an appropriate level of expertise to confirm that it is of an acceptable scientific standard.

# Peer review: Reviewer response for version 2

**DOI:** 10.21956/hrbopenres.14333.r28556

**Published:** 2021-01-06

**Authors:** Jessica McNeil

**Affiliations:** 1 Department of Cancer Epidemiology and Prevention Research, CancerControl Alberta, Alberta Health Services, Calgary, Canada

The revisions made by the authors are acceptable for indexing. I have no further comments.

Is the study design appropriate for the research question?

No

Is the rationale for, and objectives of, the study clearly described?

Partly

Are sufficient details of the methods provided to allow replication by others?

Partly

Are the datasets clearly presented in a useable and accessible format?

Not applicable

Reviewer Expertise:

Physical activity interventions and cancer survivorship.

I confirm that I have read this submission and believe that I have an appropriate level of expertise to confirm that it is of an acceptable scientific standard.

# Peer review: Reviewer response for version 1

**DOI:** 10.21956/hrbopenres.14004.r27456

**Published:** 2020-08-17

**Authors:** Brian McGuire

**Affiliations:** 1 School of Psychology, Centre for Pain Research, National University of Ireland, Galway, Galway, Ireland

This protocol outlines a planned evaluation of a supervised exercise programme for deconditioned cancer survivors.

 The following comments and queries may help to improve the protocol:

1. Given that “numerous systematic reviews demonstrate that exercise can mitigate” a number of negative outcomes in cancer survivors and improve quality of life, the rationale for evaluating this particular intervention could be more clearly articulated.
2. “Deconditioned” has not been defined or operationalised.
3. While the protocol states that this is primarily a feasibility study, the authors are also looking at evidence of treatment effect and plan to gather information to inform the sample size required for a subsequent definitive trial. Notwithstanding that this is a feasibility study, the proposed sample size of 40 requires some justification.
4. The study design involves a single arm trial with pre-, post- and 3 month follow-up. While single arm evaluations are often proposed for feasibility and pilot studies, they are inevitably weak when compared with a controlled study. Indeed, there are benefits to having a control group in a feasibility or pilot study since this can provide important information about the feasibility of recruiting and retaining a control group and about treatment effectiveness over and above the effect of "attention".
5. It would be helpful to say a little more about the background to the programme. While it is said to derive from international best practice guidelines, it would be helpful to know whether it is a pre-existing program, or building on something that already exists, or entirely new.
6. The rationale for including a health economics component (EQ-5D-5L) as a primary outcome measure should be justified, especially since there is no comparison group against which to compare costs.
7. The rationale for including prostate-specific measures focused on incontinence and sexual functioning is unclear unless there is evidence that exercise would address these complications (this was not addressed in the introduction).
8. There are some typos: The section headed “maintenance of exercise intervention” has some problems with expression within the sentence. Under “safety”, principle should be principal.

Is the study design appropriate for the research question?

Yes

Is the rationale for, and objectives of, the study clearly described?

Partly

Are sufficient details of the methods provided to allow replication by others?

Partly

Are the datasets clearly presented in a useable and accessible format?

Not applicable

Reviewer Expertise:

Psychosocial interventions for chronic physical illnesses.

I confirm that I have read this submission and believe that I have an appropriate level of expertise to confirm that it is of an acceptable scientific standard, however I have significant reservations, as outlined above.

# Peer review: Reviewer response for version 1

**DOI:** 10.21956/hrbopenres.14004.r27071

**Published:** 2020-01-10

**Authors:** Jessica McNeil

**Affiliations:** 1 Department of Cancer Epidemiology and Prevention Research, CancerControl Alberta, Alberta Health Services, Calgary, Canada

This protocol describes a one-arm intervention aimed at assessing the feasibility of a 10-week multi-modal exercise program offered to cancer survivors within the clinical setting following completion of adjuvant treatments in Ireland. Although this type of feasibility research in the health care setting is needed, some details are missing from the methods section. Furthermore, it is unsure why the authors chose to conduct a one-arm trial, when the evidence provided would be much stronger if a control comparison group (or usual care condition) was used, even if this is a feasibility study. Additional comments are provided for your consideration.

*Introduction:*

 The statements of “implementation of the programme would be a large step towards integration of exercise rehabilitation into survivorship services” is a big leap, considering that this is a one-armed pilot study to assess the feasibility of the proposed intervention. Please amend this statement to better reflect the potential impact of the proposed intervention on survivorship care.

 Please define the term “FIXCAS” at first mention.   

*Methods:*

 Why did the authors choose to conduct a one-arm study, rather than have usual care as a control comparison?

 Having a control comparison group would greatly strengthen the design of the intervention and provide more convincing findings.

 Please define what is meant by “deconditioned cancer survivors”. How will this be evaluated?

 How will it be determined whether or not the participants are “medically fit” to participate in this trial? Will the study staff or treating physician/oncologist provide medical approval? Please specify.  

 Is it the norm for patients who have completed adjuvant treatment to continue to visit the National Cancer Center in Ireland? If the patients are “discharged” from seeing their Oncologists after completing adjuvant therapy, it may be difficult to retain participants in the trial and/or convince them to go to the National Cancer Center on a regular basis to exercise.

 For the intervention, please update the Schmitz *et al*. (2010) reference to include the new ACSM guidelines publications that came out in 2019.

 Why did the authors choose to schedule supervised exercise sessions twice weekly for 60 minutes each, given that exercise recommendations are to participate in 150-300 minutes/week of physical activity? Will there be a ramp-up period to help participants adapt to this intensity and frequency of exercise? Furthermore, which components of the Theory of Planned Behavior are being used to design this intervention, and how are these components being integrated into the intervention? Please provide additional details.

 Please specify which resistance training and balance exercises will be performed and how long will the resistance exercise session last? It seems like a total of 60 minutes may not be enough to complete the proposed 30 minutes of aerobic exercise, resistance and balance exercises. A figure demonstrating a breakdown of each exercise that will be performed during the 60-minute session would be helpful.  

 Will study outcomes and habitual physical activity participation be assessed after a follow-up time period to determine whether or not the participants were able to maintain the exercise intervention and its benefits on health outcomes?

 What instrument(s) will be used to assess body composition? Please specify.

 Will the participants and health professionals be randomly selected to participate in the qualitative interviews? If not, how will the study team select the participants to participate in these interviews?

 The authors mention that the FIXCAS programme will be tailored to each individual mid-way through the Discussion section, however this is not described in the methods section. Please provide more details on how the exercise programme will be tailored to each individual and which measures will be used to tailor the intervention.

Is the study design appropriate for the research question?

No

Is the rationale for, and objectives of, the study clearly described?

Partly

Are sufficient details of the methods provided to allow replication by others?

Partly

Are the datasets clearly presented in a useable and accessible format?

Not applicable

Reviewer Expertise:

Physical activity interventions and cancer survivorship.

I confirm that I have read this submission and believe that I have an appropriate level of expertise to confirm that it is of an acceptable scientific standard, however I have significant reservations, as outlined above.
